# Utility of TMS to understand the neurobiology of speech

**DOI:** 10.3389/fpsyg.2013.00446

**Published:** 2013-07-15

**Authors:** Takenobu Murakami, Yoshikazu Ugawa, Ulf Ziemann

**Affiliations:** ^1^Department of Neurology, Fukushima Medical UniversityFukushima, Japan; ^2^Department of Neurology, Goethe-UniversityFrankfurt am Main, Germany; ^3^Department of Neurology and Stroke, and Hertie Institute for Clinical Brain Research, Eberhard-Karls-UniversityTübingen, Germany

**Keywords:** transcranial magnetic stimulation, speech perception, speech imitation, sensorimotor integration of speech, motor evoked potential, short-interval intracortical inhibition, paired-coil TMS, virtual lesion

## Abstract

According to a traditional view, speech perception and production are processed largely separately in sensory and motor brain areas. Recent psycholinguistic and neuroimaging studies provide novel evidence that the sensory and motor systems dynamically interact in speech processing, by demonstrating that speech perception and imitation share regional brain activations. However, the exact nature and mechanisms of these sensorimotor interactions are not completely understood yet. Transcranial magnetic stimulation (TMS) has often been used in the cognitive neurosciences, including speech research, as a complementary technique to behavioral and neuroimaging studies. Here we provide an up-to-date review focusing on TMS studies that explored speech perception and imitation. Single-pulse TMS of the primary motor cortex (M1) demonstrated a speech specific and somatotopically specific increase of excitability of the M1 lip area during speech perception (listening to speech or lip reading). A paired-coil TMS approach showed increases in effective connectivity from brain regions that are involved in speech processing to the M1 lip area when listening to speech. TMS in virtual lesion mode applied to speech processing areas modulated performance of phonological recognition and imitation of perceived speech. In summary, TMS is an innovative tool to investigate processing of speech perception and imitation. TMS studies have provided strong evidence that the sensory system is critically involved in mapping sensory input onto motor output and that the motor system plays an important role in speech perception.

## Introduction

Previous lesion studies provided evidence that brain areas of language production and perception are separate and independent; speech production links to the motor system of the inferior frontal area while speech perception is processed in the sensory system of the posterior temporal area in the left hemisphere (Hayward et al., 1977; Mazzocchi and Vignolo, 1979; Cappa and Vignolo, 1983; Kertesz, 1993). Recent speech models, however, have claimed that the sensory system is involved in speech production and that the motor system is activated in speech perception, suggesting that the sensory and motor systems dynamically interact in speech processing (Hickok and Poeppel, 2004, 2007; Rauschecker and Scott, 2009; Pulvermuller and Fadiga, 2010; Hickok et al., 2011). One hypothesis about language development supposed that language evolved from manual gestures, extending observation-execution matching processes to map sensory inputs onto matching motor articulation (Rizzolatti and Arbib, 1998; Corballis, 2010). This hypothesis originated from the motor theory of speech perception; speech sounds are not recognized on the basis of auditory representations, but on the basis of the motor representations that underlie speech gestures (Liberman and Mattingly, 1985). This motor theory is closely related to the concept of mirror neurons linking execution and observation for action understanding (Gallese et al., 1996; Rizzolatti and Arbib, 1998). Conversely, other evidence suggested that sensory speech perception plays an important role in modulating motor articulation at the behavioral level in health (Burnett et al., 1998; Houde and Jordan, 1998) and disease, such as stroke (Damasio and Damasio, 1980; Hickok et al., 2000). Behavioral evidence supported the existence of a close connection between speech perception and production (Galantucci et al., 2006), and neuroimaging studies revealed that neural activities in the motor and sensory speech systems are tightly coupled (Hickok et al., 2003; Wilson et al., 2004). While it is now no longer controversial that there exists a tight relation between speech perception and production, it is controversial to what extent speech perception is critically involved in articulation, and to what extent the motor representations are critically important for speech perception.

Transcranial magnetic stimulation (TMS) has widely been used in the cognitive neurosciences, including speech studies, while the most favorable method has been magnetic resonance imaging (MRI). Single-pulse TMS of the primary motor cortex (M1) can measure excitability of the corticospinal and corticobulbar tracts by motor evoked potential (MEP) amplitudes (Hallett, 2007). The theory of observation-execution matching predicts that observation of motor action leads to an increase of excitability of M1 representations specifically related to the observed action. Therefore, MEP amplitude should increase in muscles matching the observed action. Novel approaches, such as repetitive TMS (rTMS) in virtual lesion mode (Ziemann, 2010) and paired-coil focal TMS (Koch et al., 2007), can test functional relevance and task-related effective connectivity in the cortico-cortical sensorimotor speech network, respectively. Here, we present an up-to-date review of prior studies that used TMS to investigate observation-execution matching processes of speech perception and imitation.

## Measurement of cortico-bulbar excitability during speech perception

Single-pulse TMS over the hand and the face areas of M1 elicits MEPs that can be recorded by surface electromyogram (EMG) from hand and face muscles, respectively. The motor hot spot of the M1 face area is located 3.2 ± 0.5 cm lateral and 1.8 ± 0.4 cm anterior from the hot spot of the M1 hand area (mean ± SD) (Murakami et al., 2011). The elicited maximum MEP amplitude from face muscles is usually substantially smaller than the MEP amplitude from hand muscles (Devlin and Watkins, 2007). The latency of the MEP of face muscle is typically approximately 10 ms and shorter than the latency of the MEP from hand muscle (approximately 20 ms) because of the difference in conduction distance (Devlin and Watkins, 2007). Recording the MEP amplitudes offers an opportunity to test non-invasively and painlessly the excitability of the motor pathways to the target muscles, i.e., corticospinal and corticobulbar excitability (Mottonen and Watkins, 2012).

Several previous studies have reported increases of MEP amplitude of facial muscles during speech perception. Sundara et al. (2001) demonstrated that MEP amplitude increased specifically in the orbicularis oris (OO) muscle (i.e., the muscle that closes the mouth and puckers the lips when it contracts) but not in a hand muscle during viewing of lip movements articulating the phoneme /ba/ (Sundara et al., 2001). Fadiga et al. (2002) showed that MEP amplitude from the tongue increased when listening to phonemes such as /rr/ articulated from the tongue, while listening to phonemes such as /ff/, which do not involve tongue but rather lip movements did not lead to tongue MEP facilitation (Fadiga et al., 2002). These studies provide evidence for articulator specific increases of M1 excitability during viewing lip movements and listening to speech. Later, these findings were extended by showing that MEP amplitudes from the tongue increased when listening to rare words compared to frequent words, suggesting that a lexical factor influences modulation of M1 excitability (Roy et al., 2008). This lexical facilitation of the M1 tongue representation appeared 100–200 ms later than the facilitation when listening to pseudo-words, suggesting that purely articulatory vs. lexical M1 facilitation are different processes (Roy et al., 2008). Watkins et al. (2003) reported that the MEP amplitude from OO increased while there was no notable change in the MEP amplitude from a hand muscle, the first dorsal interosseous (FDI) muscle, when listening to speech and viewing speech related lip movements, supporting the notion that observation-execution matching processes link speech-related visual and auditory input in a somatotopically specific fashion to effector representation in M1 (Watkins et al., 2003).

We investigated to what extent changes in MEP amplitudes are graded with speech perception difficulty by providing subjects, while they were viewing speech-related lip movements, with variable speeds of lip movements, or while they were listening to speech, with different levels of contamination by background noise. The magnitude of MEP increase in the OO muscle was directly related to speech perception difficulty (Murakami et al., 2011), extending the notion that the observation-execution matching processes are modulated by task difficulty. This is similar to MEP facilitation of the M1 hand representation in conditions of actual hand movements which are graded by task difficulty (Datta et al., 1989; Hasegawa et al., 2001).

Recently, neurons that increase firing rate during action execution but decrease firing rate during action observation have been recorded in the ventral premotor cortex (PMv, area F5) in the macaque brain (Kraskov et al., 2009) and in the supplementary motor area of humans (Mukamel et al., 2010). These findings strongly suggest that inhibitory control is involved in action observation. To clarify the contribution of inhibitory mechanisms in M1 during observation of speech perception, we measured short-interval intracortical inhibition (SICI), a paired-pulse TMS measure of GABAAergic inhibition in human M1 (Ziemann et al., 1996). We demonstrated that SICI in the OO but not in the FDI increased when viewing lip movements related to speech (Murakami et al., 2011), supporting the recent evidence from intracortical extracellular single cell recordings that inhibitory control mechanisms are recruited, very likely to suppress unwanted motor action during action observation (Kraskov et al., 2009; Mukamel et al., 2010). In addition, greater MEP and SICI facilitations were observed from OO when viewing lip movements related to speech compared to viewing lip movements non-related to speech. These findings were supported by a functional MRI (fMRI) study, which showed only minor activation in Broca's area during viewing lip smacking movements but strong activation during viewing lip movement related to speech (Buccino et al., 2004). It seems that the motor system, including the M1 lip area and Broca's area, strongly activates through observation-execution matching processes in the human putative mirror neuron system if the observer recognizes lip movements related to speech.

In sum, the reviewed single-pulse and paired-pulse TMS studies provide consistent evidence for an observation-execution matching process in the articulatory human M1 during viewing lip movements related to speech and listening to speech. This matching process shows increase in articulatory M1 excitability that is characterized by effector specificity, task specificity, and dependence on perception task difficulty. Effector and task specific inhibitory control mechanisms are also involved to fine-tune M1 excitability appropriately and prevent from releasing articulatory action during action observation of speech-related lip movements.

## Measurement of effective connectivity using a paired-coil TMS approach

Recent neuroimaging studies provide evidence that language processing in humans occurs in a left hemispheric sensorimotor cortical network including the posterior part of superior temporal sulcus (pSTS) that functionally connects with the posterior part of inferior frontal gyrus (pIFG), i.e., the posterior part of Broca's area (Hickok and Poeppel, 2004, 2007; Buchsbaum et al., 2011; Hickok et al., 2011). A combined positron emission tomography (PET) with TMS study found that the regional cerebral blood flow (rCBF) in pIFG correlated positively with increases in excitability of the M1 lip area as measured by MEP amplitude from OO muscle while listening to speech, indicating that excitability of the M1 lip representation is directly driven by input from pIFG when listening to speech (Watkins and Paus, 2004). In addition, those authors also found that rCBF in the medial part of the left hemispheric parieto-temporal junction (Tpj), which is considered as area Spt (Hickok et al., 2003), also correlated with M1 excitability during listening to speech (Watkins and Paus, 2004). There is anatomical evidence that the pIFG receives sensory inputs from area Spt (Petrides and Pandya, 2002). This notable evidence indicates that these regional activities related to auditory speech perception correlate directly with excitability of the M1 lip area, mediated through anatomical and functional connectivity along the auditory dorsal stream (Friederici, 2009). However, it remained uncertain how these activities are linked together during auditory speech perception to map speech signals onto articulatory motor activation.

To explore effective connectivity, i.e., the causal impact of neural activity between specific brain regions and M1, a novel paired-coil TMS procedure has been introduced. Davare et al. (2008) demonstrated that the PMv → M1 effective connectivity enhanced when subjects performed a precision grasp, while this connectivity was inhibitory at rest and did not change during a power grip, suggesting that physiological interactions from PMv to the M1 hand area are specifically modulated depending on the grasp context (Davare et al., 2008). Later, these authors extended the evidence for this task-specific modulation of effective connectivity by combining paired-coil and rTMS techniques. They examined the modulation of PMv → M1 effective connectivity during preparations of two different grasp movements (precision grip vs. whole hand grasp). Enhancement of the PMv → M1 effective connectivity was specific to the upcoming grasp pattern. Disruption of activity in anterior intraparietal area (AIP) by continuous theta-burst TMS (TBS) resulted in a diminution of the task-dependent enhancement of PMv → M1 effective connectivity. This finding indicated that the AIP plays a causal role in modulating the grasp-specific effective connectivity linking PMv with the M1 hand area (Davare et al., 2010). Another study using the paired-coil TMS protocol demonstrated that connectivity linking both PMv and AIP with the M1 hand area increases when observing goal directed hand movements. The authors speculated that visual inputs from AIP might influence M1 excitability directly and, additionally, through an indirect route via PMv (Koch et al., 2010).

**Figure 1:**
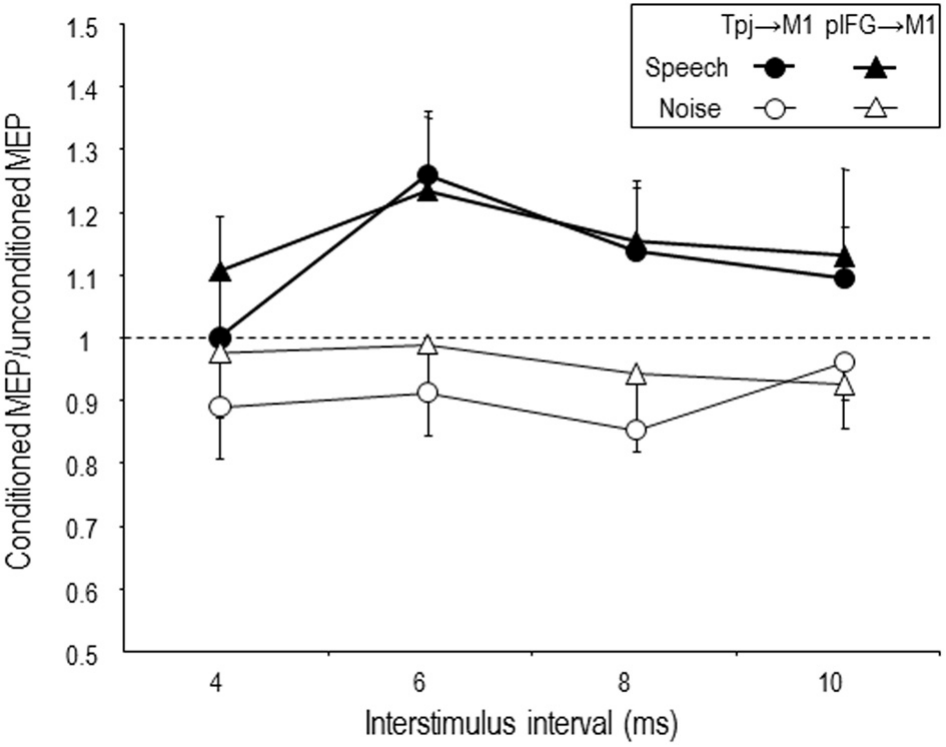
**Mean task-dependent Tpj → M1 (circle) and pIFG → M1 (triangle) effective connectivity during listening to speech (filled symbols) or to white noise (control condition, open symbols)**. Effective connectivity was studied at four interstimulus intervals between the conditioning pulse (Tpj or pIFG) and the test pulse (M1 representing to lip area). Both Tpj → M1 and pIFG → M1 effective connectivity increased during listening to speech when compared to listening to white noise. At the interstimulus interval of 6 ms, the most prominent facilitation of the effective connectivity was observed. Error bars are SEM. Figure is reproduced, with permission from Murakami et al. (2012), Elsevier.

**Figure 2:**
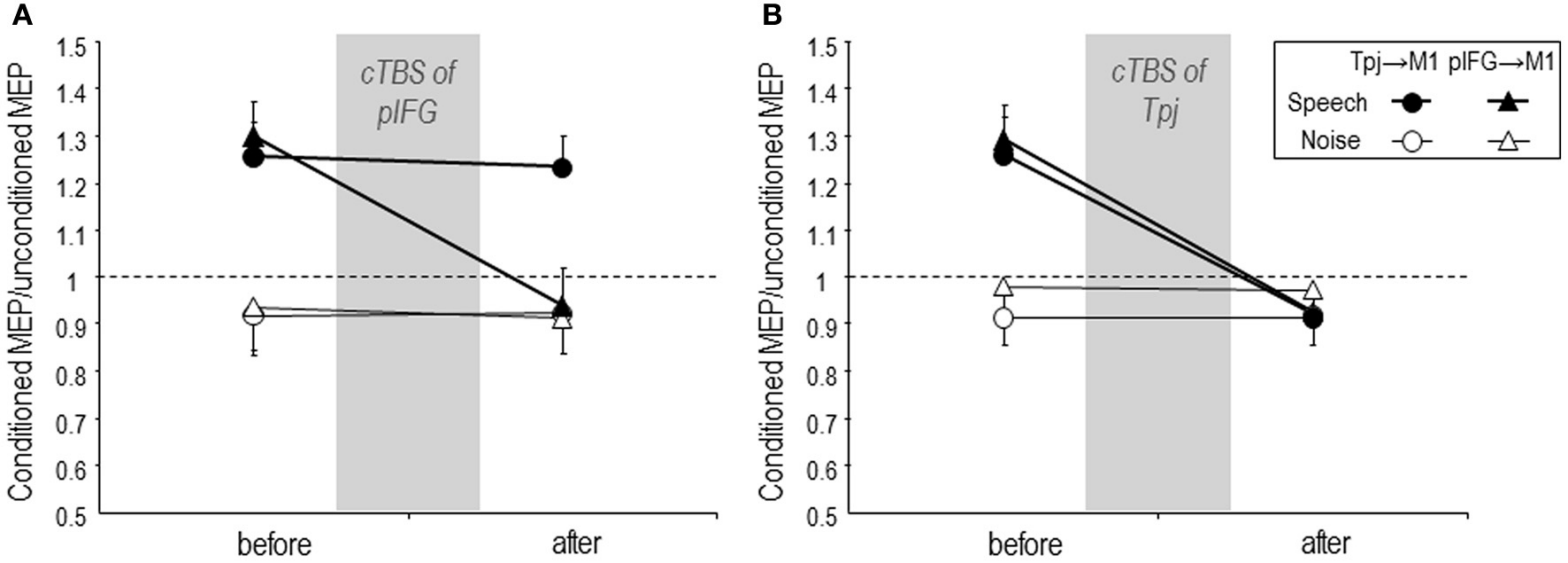
**Mean task-related Tpj → M1 (circle) and pIFG → M1 (triangle) effective connectivity during listening to speech (filled symbols) or to white noise (control condition, open symbols) before and 5 min after cTBS over the pIFG (A) or over the Tpj (B). (A)** cTBS of the pIFG abolished speech task-related increase of pIFG → M1 effective connectivity when listening to speech but did not modulate Tpj → M1 effective connectivity. **(B)** cTBS of the Tpj abolished the task-dependent increase of both Tpj → M1 and pIFG → M1 effective connectivity. Error bars are SEM. Figure is reproduced, with permission from Murakami et al. (2012), Elsevier.

Recently, we have reported modulation of effective connectivity from speech related regions to the M1 lip area during auditory speech perception using the paired-coil TMS approach (Murakami et al., 2012). In accordance with the previous studies (Davare et al., 2008, 2010; Koch et al., 2010; Catmur et al., 2011), we performed paired-coil TMS when listening to speech or listening to white noise. The conditioning TMS pulse was delivered to pIFG or Tpj of the left hemisphere, respectively. The test pulse was applied over the left M1 lip area to elicit MEPs in the right OO muscle. An fMRI-guided TMS neuronavigation system was used to define individual stimulation sites precisely. The individual coordinates for TMS targeting were determined as the maximum individual fMRI activations in close proximity to the pIFG and the Tpj as defined in the group fMRI data in order to take into account inter-individual differences (Andoh and Paus, 2011). The conditioning pulse was applied prior to the test pulse and four different interstimulus intervals (ISI; 4, 6, 8, 10 ms) were examined. An index of the effective connectivity of pIFG → M1 and Tpj → M1 was calculated by the ratio of MEP amplitude elicited by paired-pulse TMS (conditioned MEP) over the MEP amplitude elicited by the test pulse alone (test MEP). Effective connectivity of both pIFG → M1 and Tpj → M1 increased at the ISI of 6 ms when listening to speech compared to white noise (Figure 1). This finding provides direct evidence for a causal role of task-driven neural activity in the left pIFG and Tpj for sensorimotor integration of speech perception. Furthermore, we applied continuous TBS (cTBS) to disrupt neuronal activity in the pIFG and Tpj, respectively, for testing the critical role of this activity for the observed task-dependent modulation of pIFG → M1 and Tpj → M1 effective connectivity. CTBS of pIFG abolished the task-dependent increase of pIFG → M1 but not Tpj → M1 effective connectivity when listening to speech (Figure 2A), while cTBS of Tpj led to disappearance of the task-dependent increases of both effective connectivities (Figure 2B). Similarly, the previous paired-coil TMS experiment in combination with a virtual lesion cTBS protocol demonstrated that disruption of AIP activity by cTBS reduced the task-dependent PMv → M1 effective connectivity during preparation of hand movements to grasp objects (Davare et al., 2010). These convergent findings provide direct evidence that the Tpj and AIP in the parietal cortex serve as important nodes of a sensorimotor interface that is situated at a hierarchically high level, integrating auditory and visual inputs into motor outputs through but also bypassing the pIFG and PMv (Hickok and Poeppel, 2004, 2007; Murakami et al., 2012). On a clinical perspective, conduction aphasia, i.e., normal speech comprehension but difficulty in repeating the perceived words, typically results from lesions of Tpj in the left hemisphere (Buchsbaum et al., 2011). We did not perform behavioral testing in our study (Murakami et al., 2012), but it would be predicted that disruption of the pIFG → M1 and, Tpj → M1 effective connectivities after virtual lesion of Tpj by cTBS would lead to an increases of phonemic errors during speech repetition.

## Modulation of speech imitation and perception by repetitive and event-related TMS

To investigate the causal involvement of the neural activity of specific brain regions in a specific behavioral task, TMS can be used in the so called “virtual lesion mode” (Ziemann, 2010). By applying rTMS or event-related TMS over a certain brain area, the activity of the stimulated network is disrupted or facilitated, which should result, respectively, in decline or improvement of behavioral performance if this network is relevant for the performed task. Two prior TMS virtual lesion studies provided evidence for the role of sensorimotor integration during action observation. Heiser et al. (2003) applied rTMS over the left pIFG or occipital cortex and recorded imitative finger performance when observing finger key presses (imitative performance) or spatially cued executive finger movements (non-imitative performance). They showed a significant impairment of the imitative performance but not the non-imitative performance when applying rTMS over the left pIFG. In contrast, rTMS of the occipital cortex did not lead to modulation of imitative or non-imitative performance. These results indicated that the left pIFG plays an essential role in finger movement imitation (Heiser et al., 2003). The other study performed cTBS over the left pIFG or posterior parietal cortex (PPC) and measured reaction time of compatible versus incompatible finger movements. CTBS of the left pIFG significantly delayed reaction time of compatible but not incompatible finger movements, while cTBS of the left PPC did not alter either movement, suggesting that the left pIFG selectively modulates compatible movements (Catmur et al., 2009). Those prior reports provide evidence that activity of the left pIFG is involved in motor imitation of the observed action.

We applied TBS to induce a temporal “virtual lesion” over the left pIFG to investigate to what extent speech imitation of auditory-presented sentences can be modulated (Restle et al., 2012). In a main experiment, we delivered three patterns of TBS protocols (iTBS; intermittent TBS, cTBS; continuous TBS, imTBS; intermediate TBS) to the left pIFG using an fMRI-guided neuronavigation system and evaluated accuracy of speech imitation of short sentences of a foreign language (German native speakers speech-imitated Japanese short poem sentences) before and after TBS. The three TBS protocols were chosen, because, when applied over M1, iTBS resulted in increase, cTBS in decrease, and imTBS in no significant change of excitability (Huang et al., 2005). The foreign language task was chosen for investigation of sensorimotor integration of speech imitation along the auditory dorsal stream because foreign language provides phonological but not lexical or semantic information which are processed in the ventral stream (Hickok and Poeppel, 2004). We found that accuracy of speech imitation significantly improved after excitability-enhancing iTBS and, to a lesser extent, after imTBS, while no notable change was observed after cTBS of the left pIFG. For control, iTBS was applied over the left middle occipital gyrus (MOG), which is not concerned with sensorimotor integration of auditory speech processing. Imitation accuracy remained unchanged after iTBS of MOG. These findings indicate a causal role of the left pIFG as a part of the auditory dorsal stream in sensorimotor integration of phonological perception to matched articulation for speech imitation. Speech imitation represents a beneficial training strategy not only for acquiring new languages but also as a therapeutic approach in speech-impaired individuals (Lee et al., 2010). Combining speech imitation with facilitatory effects of non-invasive brain stimulation over the left pIFG, hence, may notably extend the potential for speech training and therapy of aphasia after stroke (Baker et al., 2010; Fridriksson et al., 2011; Schlaug et al., 2011; Szaflarski et al., 2011).

Finally, several studies applied rTMS over motor cortical areas to examine a causal role of the motor system in speech perception. Meister and colleagues applied inhibitory low-frequency rTMS over the left premotor cortex and tested syllable identification. The accuracy of syllable identification decreased after rTMS over the left premotor cortex, while control tasks (color and tone discrimination) were not impaired (Meister et al., 2007). Sato and colleagues demonstrated that low-frequency rTMS over the left PMv resulted in slower phoneme discrimination in a phonemic segmentation task when compared to sham stimulation (Sato et al., 2009). These two studies provide evidence for the causal role of the left-hemispheric premotor areas in syllable recognition.

Mottonen and Watkins (2009) examined syllable discrimination before and after low-frequency rTMS to disrupt excitability of the M1 lip area. Inhibitory rTMS of the M1 lip area impaired discrimination of lip-articulated syllables, whereas discrimination of two sounds that are not related to lip movements were not affected by the stimulation, nor was discrimination of the lip-related syllables compromised when rTMS was applied to the M1 hand area (Mottonen and Watkins, 2009). Recently, these authors have investigated to which extent rTMS modulates miss match negativity (MMN) responses to phonetic changes in auditory vowel sound by combining TMS with electroencephalogram (EEG). RTMS-induced disruption of the M1 lip area decreased the MMN responses to infrequent phoneme, while the MMN amplitudes remained unchanged by rTMS of the M1 hand area. Furthermore, disruption of the M1 lip area by rTMS did not lead to modulation of the MMN related to changes in non-verbal piano tones (Mottonen et al., 2013). Those findings provide further evidence that the articulatory representation in M1 contributes to the recognition of speech phonemes (Watkins et al., 2003). In another TMS study that investigated the functional role of the motor system in speech recognition, participants had to identify speech sound, which consisted of either lip-articulated or tongue-articulated syllables after event-related double-pulse TMS of the M1 lip vs. M1 tongue representation. Stimulation of the M1 lip area selectively facilitated identification of lip-articulated syllables, while TMS of the M1 tongue area selectively improved identification of tongue-related syllables (D'Ausilio et al., 2009). These authors extended this finding by showing that the enhancement of speech recognition by event-related TMS of articulatory M1 was present in a noisy environment but not in a noise-free condition (D'Ausilio et al., 2012). Using the same TMS method, D'Ausilio and colleagues have investigated the functional role of laryngeal M1 in vocal pitch discrimination. Stimulation of the M1 representing larynx led to faster recognition of vocal pitch discrimination compared with stimulation of the M1 lip area (D'Ausilio et al., 2011). Strikingly, these studies provide convergent evidence that the M1 representations of the articulatory musculature contribute to recognition of speech perception, in accord with the motor theory of speech perception (Liberman and Mattingly, 1985).

These approaches employing rTMS or event-related TMS to disruptor enhance neural activity in speech related cortical areas have elucidated that the sensory system is involved in mapping sensory perception onto motor articulation and that the motor system plays a causal role in speech perception. The evidence from TMS studies supports recently developed speech models that the sensory system primarily modulates speech production (Rauschecker and Scott, 2009; Hickok et al., 2011) and the motor system also provides feedforward information onto the sensory system to support speech perception (Rauschecker and Scott, 2009; Pulvermuller and Fadiga, 2010; Hickok et al., 2011).

When comparing TMS to neuroimaging methods, e.g. fMRI and EEG, TMS provides several advantages and disadvantages: paired-pulse TMS of M1 allows investigation of task-related changes in intracortical inhibition and facilitation (Murakami et al., 2011) while investigation of neural inhibition is still not unambiguously possible with fMRI. Paired-coil TMS allows determination of task-related modulation of effective connectivity (Murakami et al., 2012) that should be viewed as a complementary approach to analysis of effective connectivity from fMRI and EEG data. Finally, repetitive and event-related TMS allow testing of causality between activity in neural circuits and behavioral task performance (D'Ausilio et al., 2009; Mottonen and Watkins, 2009; Restle et al., 2012), which is not possible with fMRI or EEG. TMS application to one region typically modulates not just the directly stimulated region but also other brain regions that are functionally or anatomically connected with the stimulated site. Combining TMS with neuroimaging techniques can turn this potentially weak point into advantage and help us to understand the comprehensive brain networks related to speech perception and imitation (Mottonen and Watkins, 2012).

In conclusion, this review demonstrates that TMS has developed into an extremely useful tool to investigate the role of the sensorimotor system in speech perception and imitation. Innovative protocols, such as a paired-coil focal TMS, repetitive and event-related TMS in virtual lesion mode, offer opportunities to examine causality between neural activity in sensori-motor cortico-cortical networks and speech perception and imitation. In addition, rTMS interventions may provide beneficial effects for speech training or rehabilitation of aphasic patients.

### Conflict of interest statement

The authors declare that the research was conducted in the absence of any commercial or financial relationships that could be construed as a potential conflict of interest.
